# Polyamines and Physical Activity in Musculoskeletal Diseases: A Potential Therapeutic Challenge

**DOI:** 10.3390/ijms24129798

**Published:** 2023-06-06

**Authors:** Letizia Galasso, Annalisa Cappella, Antonino Mulè, Lucia Castelli, Andrea Ciorciari, Alessandra Stacchiotti, Angela Montaruli

**Affiliations:** 1 Department of Biomedical Sciences for Health, University of Milan, 20133 Milan, Italy; letizia.galasso@unimi.it (L.G.); annalisa.cappella@unimi.it (A.C.); lucia.castelli@unimi.it (L.C.); andrea.ciorciari@unimi.it (A.C.); alessandra.stacchiotti@unimi.it (A.S.); angela.montaruli@unimi.it (A.M.); 2 U.O. Laboratorio di Morfologia Umana Applicata, I.R.C.C.S. Policlinico San Donato, San Donato Milanese, 20097 Milan, Italy; 3 I.R.C.C.S. Ospedale Galeazzi-Sant’Ambrogio, 20157 Milan, Italy

**Keywords:** polyamines, spermidine, spermine, autophagy, mitophagy, aging, sarcopenia, physical activity, exercise

## Abstract

Autophagy dysregulation is commonplace in the pathogenesis of several invalidating diseases, such as musculoskeletal diseases. Polyamines, as spermidine and spermine, are small aliphatic cations essential for cell growth and differentiation, with multiple antioxidant, anti-inflammatory, and anti-apoptotic effects. Remarkably, they are emerging as natural autophagy regulators with strong anti-aging effects. Polyamine levels were significantly altered in the skeletal muscles of aged animals. Therefore, supplementation of spermine and spermidine may be important to prevent or treat muscle atrophy. Recent in vitro and in vivo experimental studies indicate that spermidine reverses dysfunctional autophagy and stimulates mitophagy in muscles and heart, preventing senescence. Physical exercise, as polyamines, regulates skeletal muscle mass inducing proper autophagy and mitophagy. This narrative review focuses on the latest evidence regarding the efficacy of polyamines and exercise as autophagy inducers, alone or coupled, in alleviating sarcopenia and aging-dependent musculoskeletal diseases. A comprehensive description of overall autophagic steps in muscle, polyamine metabolic pathways, and effects of the role of autophagy inducers played by both polyamines and exercise has been presented. Although literature shows few data in regard to this controversial topic, interesting effects on muscle atrophy in murine models have emerged when the two “autophagy-inducers” were combined. We hope these findings, with caution, can encourage researchers to continue investigating in this direction. In particular, if these novel insights could be confirmed in further in vivo and clinical studies, and the two synergic treatments could be optimized in terms of dose and duration, then polyamine supplementation and physical exercise might have a clinical potential in sarcopenia, and more importantly, implications for a healthy lifestyle in the elderly population.

## 1. Introduction

Skeletal muscle is one of the most abundant tissues in humans. It constitutes more than 30–40% of the total body mass and extends its vital functions from posture, movement, and breathing to fundamental regulatory and metabolism functions of protein, lipid, and glucose [1]. Indeed, muscles are crucial to maintain thermogenesis and body temperature [2]. Moreover, skeletal muscles are a key site for glucose uptake from the blood and deposition as glycogen; therefore, they are targets of insulin via insulin-like growth factor 1 [3]. Some current evidence considers skeletal muscle an “endocrine organ” to all effects. As a response to exercise, it interfaces with the whole body through the secretion of myokines, soluble cytokines, or chemokines [4]. Specific myokines, such as irisin, interleukin 6 (IL-6), and insulin growth factor 1 (IGF-1) not only drive autocrine anabolism in muscles, but also mediate anti-inflammatory response and bone and adipose tissue crosstalk via receptor-mediated paracrine/endocrine action [5,6]. In primary myocytes, more than hundreds of myokines have been characterized in humans and rodents although their biological function has been described for only 5% of these [4,7].

Remarkably, joints and bones are crucial organs among those with which the skeletal muscle interacts. In addition, their strict connection is not only necessary for movements but is of utmost importance for influencing their reciprocal metabolism, which is deeply mediated by mutual biochemical and molecular mechanisms [8]. The bone cell-derived proteins named osteokines (such as fibroblast growth factors, osteocalcin, nuclear factor kappa B receptor activator ligand (RANKL), IGF-1, or small molecule-like prostaglandin E2) influence the bone and muscle homeostasis directly or indirectly [9,10]. The bidirectional changes occurring between these tissues, which often extend to the adipose depots, are maintained by common endocrine soluble factors, as recently reviewed by Kirk et al., 2020 [11]. Moreover, to connect muscles to other tissues, such as bone, myotubes produce extracellular vesicles called exosomes in the extracellular medium in vitro or in body fluids and serum [12,13]. In fact, exosomes, budded from the sarcoplasmic membrane or from late endosome-multivesicular bodies, may transfer a multitude of mediators as myokines, muscle-specific mRNAs, and lipids crucial for muscle function and regeneration [14,15,16].

In vertebrate, the healthy skeletal muscles own a complex architecture that comprehends different cell types, including polynucleated myofibers, quiescent satellite cells, fibroblasts, adipocytes, and macrophages. Several myofibers group together in larger units, called fascicles, which are further associated with the total muscle [17]. Moreover, muscles are enveloped by intramuscular connective sheets, surrounded by the extracellular matrix, which is the site that hosts blood supply and innervation [18,19]. This highly differentiated system is based on a functional unit named sarcomere, which is the site of force generation characterized by actin and myosin filaments cross-linked with other structural proteins able to fix calcium ions [20]. Moreover, at subcellular level, adult myotubes contain a set of interrelated organelle-like mitochondria, sarcoplasmic reticulum, and lysosomes, which collaborate to produce energy, drive calcium flux, and dismantle dysfunctional proteins and cellular debris.

The presence of different myosin heavy chain (MYH) isoforms in myofibers permits the identification of different muscles in mouse and humans [21]. In rodents, muscles are classified according to four types of fibers: Slow type 1 or fast types 2A, 2X, and 2B. Conversely, human muscles contain three types of fibers: Slow type 1 and fast types 2A and 2X. Currently, in addition to a consolidated older classification, single fiber proteome and single nucleus transcriptome analysis characterize sarcoplasmic proteins and mitochondria for each fiber and measure oxidative metabolism and regeneration [22]. Type 2A and type 2X fibers, based on oxidative metabolism, contained the major number of mitochondria, while the fast highly glycolytic type 2B fiber contains the lower number [23].

Mitochondria are scattered in sarcomere I band within glycolytic fibers, while in aerobic fast fibers they accumulated in both I and A bands [24]. However, a mixture of different myofibers in human skeletal muscles characterizes the adaptability of fast-twitch type 2 fibers, and the abundance of dystrophin and integrin in the myotendinous junction in type 2X fibers [25].

During aging, skeletal muscles face a multitude of structural and functional changes which progressively determine a loss in mass and strength. The cellular alteration is one of the main consequences in terms of the number of muscle fibers and their cross-sectional area [26]. Since satellite cells, the cells appointed to self-renew, are reduced in number, adipocytes and the fibrous connective tissue replace myofibers, while the paucity of elastic fibers affect adaptability [27,28]. Quiescent satellite cells rely on glycolytic metabolism; however, in response to injury or excessive loading, they use an oxidative metabolism even if their regenerative potential and number dramatically decrease in aging [29]. Remarkably, altered mitochondrial biogenesis in aged muscle leads to altered muscle repair [30]. At an early stage of aging, muscle composition changes, and type 2 fibers dramatically decrease in mice [31].

Abnormal extracellular matrix expansion and collagen I infiltration impair motility and regeneration [32]. In addition, scarce blood supply and degenerated neuromuscular junction characterized aged muscles, contributing to weakness and immobility [33]. Intramuscular adipose tissue increases in advancing age, along with resident macrophages, mainly type M2 in mice and human muscles [34]. However, low chronic grade of systemic inflammation affects muscle performance and is implicated in aging sarcopenia [35]. All of the above-mentioned changes contribute to the determination of the structural and functional deterioration occurring in skeletal muscle during aging, leading progressively to the resulting phenotype characterizing sarcopenia, and thus to the decrease in mobility, the loss of independence, and the increase in the risk of other morbidities in bones and joints [26]. Similar to skeletal muscle, the human skeleton experiences changes during life and aging. Indeed, although bone is the hardest tissue in the body, it undergoes complete remodeling during the lifespan, approximately every 10 years [36]. Long bones, the fundamental components of mobile synovial joints, consist of highly specialized trabeculae surrounding the bone marrow cavity, enveloped by a connective layer rich in vessels and nerves. Constant changes in thickness and mineral density in bones are the result of the remodeling process, which is the balance between osteoblasts (bone formation), osteoclasts (bone resorption), and their transition to quiescent osteocytes [37]. These last cells, estimated to be 42 billion in human skeleton, represent a crucial source of multiple endocrine signals, mainly cytokine receptor activator of RANKL driving osteoclast formation [38,39]. In postmenopausal women, the progression of life span and reduced estrogenic signaling cause the loss of trabecular bone thickness and mineral density and is predisposed to fractures and hospitalization [40]. Osteoclast overactivation triggers osteoporosis, and degenerative inflammation in articular cartilage is predisposed to disease-like osteoarthritis and autoimmune rheumatic arthritis in aging sarcopenia [41].

Since muscle and bone share genetic, lifestyle, and hormonal determinants, the multifactorial and complex pathophysiology and etiology of osteoporosis and sarcopenia have a synchronic relationship [42]. Recent studies highlighted a correlation between body composition, muscle strength, and bone mineral density [43]. Particularly, He et al. (2016) found that osteoporosis and sarcopenia are frequently concomitant in older women, supporting the growing evidence, not without caution, that healthy bone is progressively impaired with advancing sarcopenia stages [44]. Specifically, muscle and bone mass can be modulated by multiple factors, their dysfunctional alterations due to common environmental stimuli often co-exist, and the clinical consequences surpass the musculoskeletal system [6], as we will see in the next paragraphs.

Highly differentiated biological systems, such as skeletal muscles and bones depend on a biological environment where protein anabolism and catabolism are balanced.

In both systems, metabolic homeostasis is maintained by autophagy, a crucial housekeeping “recycling” process able to dismantle dysfunctional macromolecules and organelles [45].

Moreover, the quality control of mitochondria and other damaged macromolecules is one of the essential mechanisms for maintaining the cellular homeostasis [46]; therefore, this applies to muscle cells. In fact, the selective autophagy occurs to specifically remove damaged and dysfunctional organelles whose excessive rates are hallmark of sarcopenia and correlated musculoskeletal diseases, including bone metabolic disorders [47,48]. This is a crucial point since an appropriate induction and normal regulation of autophagy by genetic, nutritional, and pharmacological treatments can alleviate age-related diseases, and thus extend longevity [49].

Currently, there is no effective pharmacological therapy to alleviate sarcopenia [50], whereas the long-time treatment of age-associated bone metabolic disorders, osteoarthritis, and rheumatoid arthritis based on corticosteroids is associated with negative side effects [51]. In addition, bisphosphonates are not resolutive for bone mineral density in osteoporosis [52]. On the one hand, pharmacological treatments can have negative effects in addition to a proper autophagy regulation. On the other hand, some natural compounds found in health-related foods can instead safely induce appropriate autophagy. Among these natural compounds, polyamines represent autophagy-stimulating molecules that have attracted great attention in the last decade. Another interesting and safe modulator that was recently identified as an autophagy inducer in vivo is physical exercise [53,54,55,56,57,58,59]. Indeed, proper tuning of autophagy and mitophagy extent by selected “modulators”, such as safe nutraceuticals, for instance, polyamines and physical exercise, are an attractive emerging objective to limit aging-induced muscle atrophy [60,61], which is the major focus of the present narrative review.

To provide insights on this emerging topic, this narrative review first focuses on the background of sarcopenia and age-related degenerative and musculoskeletal diseases. Then, we focus on the pathways for autophagy and mitophagy in healthy striated skeletal muscle and their defects in musculoskeletal diseases. Finally, we present the latest in vitro and in vivo studies on natural polyamines, mainly spermidine, in age-related musculoskeletal diseases. Finally, in the last paragraph, few available data present in the literature and from recent in vivo studies that focus on the main objective of this narrative review are provided and discussed, which is the possible role of polyamines and exercise, alone or combined, as autophagy inducers on muscle for ameliorating age-related musculoskeletal disorders, such as sarcopenia. Herein, we intentionally did not consider cancer, cachexia, autophagy in inherited rare myopathies, and neurological disorders in critically ill patients since they have already been addressed in recent reviews [62,63,64,65].

## 2. Sarcopenia and Osteosarcopenia, Unavoidable Signs of Aging

Sarcopenia, the progressive decline of muscle mass and strength in aging, affects all mammals, including humans, and has been clinically defined by the consensus of European Working Group on Sarcopenia in Older People 2 (EWGSOP2) in 2019 [66].

Senescence in muscles is not only strongly associated with the thinning of fibers, but also with proinflammatory and oncogenic factors concurring with sarcopenia [67].

Sarcopenia consists of persistent muscle atrophy, which is the gradual loss of mobility due to the imbalance between protein synthesis and degradation in myofibers [68]. Specifically, myofibers lose the capability to adapt to the metabolic requirements and accumulated excessive debris due to the overall decrease in proper protein degradation process [69]. In the elderly, muscle composition changes and fast-twitch type 2 fibers shift to slow-twitch type I fibers, as recently confirmed by proteomic analysis [70].

However, since primary sarcopenia is strictly associated with aging; therefore, secondary sarcopenia is due to cancer, obesity, and excessive sedentary [71].

The prevalence of sarcopenia is equally distributed in male and female humans [72], even if some authors reported that males are more susceptible than females [73].

In addition to primary and secondary sarcopenia, a new geriatric syndrome linked to sarcopenia has been recently described and named as osteosarcopenia.

Hirschfeld et al. (2017) referred to “osteosarcopenia” as the association and concurrent presence of bone conditions, such as osteoporosis/osteopenia and sarcopenia, which is considered as a new syndrome in the elderly [74]. Moreover, recent studies associated osteosarcopenia with obesity and insulin resistance as a high-risk factor to falling, loss of balance, and fractures with dramatic impact on the quality of life [75,76,77,78]. This might suggest the role of a third player which is fat. In fact, its infiltration can be found within the bone marrow in osteoporotic bones and located within myofibers (intramyocellular fat) in sarcopenic muscle [79].

Impaired muscle metabolism, glucose and insulin response, and reduced markers of insulin cascade, such as IGF-1 and AKT kinase, accelerated osteosarcopenia in AKT KO mice [80].

A recent study, undertaken in more than 5900 subjects without overt cardiovascular diseases, suggests that osteosarcopenia is an important predictor element for coronary artery calcification in asymptomatic adults [81].

The pathogenesis of sarcopenia/osteosarcopenia is multifactorial and determined by the same mechanisms involved in aging and senescence in humans and rodents [82,83]. They include reduced physical activity, low-grade of chronic inflammation, oxidative stress, and disrupted autophagy.

Understanding of the mechanisms of the pathogenesis of sarcopenia and related diseases is crucial to provide preventive or therapeutic measures, considering that a resolutive senolytic therapy is lacking [30,35,62,84,85].

## 3. Autophagy in Osteosarcopenia

Skeletal muscle mass and strength are largely dependent on the balance between protein anabolism and proteolysis. Major players of anabolism are hormones, such as insulin and testosterone, and muscle catabolism is mainly driven by different proteolytic systems, such as ubiquitin-proteasome and autophagy.

The heterogeneity in myofibers’ composition and metabolism in different skeletal muscles has a drastic impact on their activity [62]. In eukaryotes, the most important molecular pathway to provide energy for maintaining muscle mass is the adenosine monophosphate-activated protein kinase (AMPK) that balances ATP production. Moreover, AMPK influences mitochondria growth and dynamic in muscles according to metabolism [86]. In response to intermittent glucocorticoids, AMPK became phosphorylated and stimulates the transcription of peroxisome proliferator-activated receptor-gamma coactivator (PGC-1 alpha) and the production of new mitochondria [87]. In mammals, mTOR-complexed proteins called mTORC1, consisting of multiple proteins, such as *raptor* and *deptor*, regulate extracellular signals and oxygen distribution. mTORC1 is upstream regulated by insulin and IGF-1, activating a cascade of events to maintain proper muscle mass. Remarkably, mTOR signaling is inhibited by specific atrophy-inducing genes called “atrogenes” [88], which are crucial in rodents, while their role in humans is still debated.

Members of Forkhead box O family (FOXOs) are the most widely known transcription factors downstream to AKT/IGF-1/insulin pathway and their modulation is necessary to maintain skeletal muscle mitochondria and calcium signaling [89]. Indeed, if FOXOs are translocated to the nucleus on induction of AMPK, then they are sustained in muscle atrophy. However, if phosphorylated, then they are excluded by the nucleus, and thus become inactive [90]. FOXO activity may be regulated by several enzymes, such as kinases (for example, AKT), acetylase or deacetylase, or by transcription factors (such as PGC-1 alpha and beta) and glucocorticoid receptor. In humans, four types of FOXOs have been identified, in which FOXO1 and FOXO3 are involved in muscle atrophy, modulating the transcription of autophagy genes and abnormal autophagy [91].

All the above-mentioned molecular pathways modulate autophagy as we will see in the following paragraphs, and if not properly regulated, they might cause an impaired autophagy until an escalating decline of muscle and bone mass and function, which characterizes osteosarcopenia.

**Figure 1:**
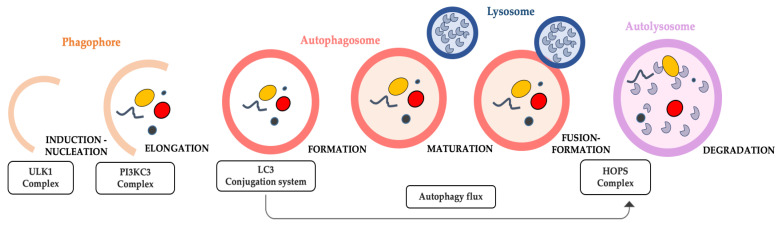
Schematic overview of the progression of autophagy. Once the induction and nucleation of phagophore have been completed thanks to the activity of ULK1 and PI3KC3 complexes, the LC3 system conjugates the sequestering membrane of initial phagophore and controls its elongation and expansion until its closure, and then the subsequent development of the next form takes place, which is the autophagosome. The contribution of LC3 complex continues for the majority of the autophagic flux, or rather until the autophagosome maturation occurs as it is anchored to its membrane. Its dissociation from the autophagosome membrane will occur upon the latter fuses with the lysosome, and thus form the autolysosome. Finally, in the autolysosome, the content (to remove dysfunctional organelles, proteins, etc.) is included, which has been gathered and closed within the initial phagophore (and thus within the following autophagosomic form) and lysosomal enzymes are released from the lysosome during its fusion with the autophagosome. Here, in the autolysosome, the autophagosome content is now degraded by the lysosomal enzymes, and thus the autophagy is concluded.

Macroautophagy, simply called here autophagy, is a “dismantling” catabolic process, highly conserved in species, from yeast, flies, worms to vertebrates. It is a critical physiological degradation process that primarily acts on damaged cells and dysfunctional organelles to recycle them, providing energy for nascent cells [92]. Dysregulated autophagy is attributed to the apparent age-related accumulation of damaged cellular components, such as defective mitochondria [69]. Autophagy process consists of almost five steps, which are regulated by specific autophagy related genes (ATG). They are induction, followed by nucleation, expansion, fusion with lysosome, cargo degradation in mature autolysosome, and finally the recycling phase (Figure 1).

Remarkably, each step is influenced by signaling molecules used as specific biomarkers. The starting phase of autophagy and autophagosome production is initiated by Unc51-like autophagy activating kinase 1 (ULK1), a serine/threonine-specific protein kinase [93]. ULK1 is the very first autophagy-specific complex, which induces the formation of the double membrane phagophore that will become later an autophagosome after the process of elongation [94]. This complex includes ULK itself in partnership with three other proteins: Autophagy-related protein 13 (Atg13) and 101 (Atg101), and focal adhesion kinase family interacting protein of 200 kDa (FIP200) [95]. Following induction, autophagy-specific class III phosphatidylinositol 3-kinase complex I (PtdIns3K-C1) is the next key player in nucleation and autophagosome formation. This second complex comprehends different subunits consisting of PIK3C3, PIK3R4, BECLIN1, and ATG14 and converts phosphatidylinositol (PI) to phosphatidylinositol 3-phosphate (PtdIns3P) through phosphorylation [96].

The ULK1 complex is inhibited and activated by the mammalian target of rapamycin complex 1 (mTORC1) and AMP-activated protein kinase (AMPK), respectively [97]. When mTORC1 is upregulated, as in conditions of high glucose levels, it prevents autophagy initiation by phosphorylating ULK1Ser757 of the ULK1 complex and by disrupting the interaction between the latter and its activator (AMPK) [98]. On the contrary, if AMPK is activated, as in the response to low glucose levels or to energetic stress, it induces autophagy [99]. Indeed, when activated, AMPK inhibits mTORC1, stimulates ULK1 kinase through phosphorylation of ULK1Ser317 and Sr777 [98] and Ser555 [100], as well as triggers FOXO3, upregulating the expression of muscle specific atrophy-induced genes [101,102]. In particular, this excessive dysregulated event in skeletal muscle can culminate in protein degradation, and thus determines atrophy [103].

The antiapoptotic protein BCL2 seems to be an important modulator of Class III PtdIns3K complex. Particularly, BCL2 is associated with BECLIN1 in normal conditions, preventing the interaction between the latter and PIK3C3. BCL2 dissociates from BECLIN1 only in the case of stress, allowing for the activation of the complex, and thus autophagy. Repression and activation of autophagy via enhancement or inhibition of BCL2/BECLIN1 interaction is modulated by a variety of proteins and compounds [104]. In addition, phosphorylation of BECLIN1 seems to be the key of this mechanism, as recently well-reviewed by Menon et al. [105].

The completion of autophagosome is characterized by the lipidated form of microtubule-associated protein 1A/1B-light chain 3 (LC3B-II), homologous of ATG8 in yeast. This crucial point of autophagy is regulated by the transcription factor EB (TFEB), which activates the shift in the nucleus and promotes the biogenesis of autophagosome and autolysosome [106].

The final step of autophagy is controlled by the sequestosome protein (p62/SQSTM). The extent of autophagy is defined by the measure of the proper autophagic flux, indicated by p62/SQSTM1 levels that increase when autophagy is aberrant in muscle atrophy [107,108].

Finally, the homotypic fusion and protein sorting (HOPS) complex (PLEKHM1 and EPG5) allows for the lysosomal and autophagosome fusion by binding the nucleoside triphosphate enzyme (RAB7) of the lysosome and the LC3B-II of the autophagosome until the fusion and secretion of lysosomal hydrolase enzymes.

Morphologically, at ultrastructural level, autophagy is characterized by autophagosomes and their fusion with lysosomes to form autophagolysosomes, peculiar double membrane vacuoles entrapping cellular debris, or disrupted organelles [109]. Recent evidence indicates that in muscles, existing membranes derived from autolysosomes are reused to produce new active lysosomes, and this reformation process requires membrane-bound phosphoinositides (PI4P and PI(4,5)P) [110].

Indeed, in muscle specific autophagy, inhibition affects its strength influencing the quality of life or the lifespan in human and mice [111]. Both imperfect or excessive activation of autophagy may contribute to the abnormal clearance of intracellular aggregates in skeletal muscle, leading to the atrophy of skeletal muscle, muscle weakness, or muscle fiber degeneration [112].

On the contrary, an appropriate induction is important for attenuating sarcopenia and other myopathies, resulting from dysfunctional autophagy [113]. Furthermore, impaired autophagic processes are related to many pathological conditions, such as diabetes mellitus, dystrophy, neurodegenerative disorders, as well as liver and heart diseases [114]. Moreover, autophagy reactivation could improve age-associated myofiber degeneration and mitochondrial dysfunction [111,115].

Notably, in healthy adult skeletal muscles, the intense aerobic metabolism and the presence of multiple nuclei and organelles in a single myofiber make autophagy fundamental. Basal autophagy is sustained, independent from starvation, to maintain the quiescent status of the satellite cells, that can provide new myofibers in the damaged area [116], and to prevent senescence [117]. Specifically, autophagy-induced satellite cells activation has been reported to act on the early compensatory regeneration of dystrophic muscles [118]. Autophagy contributes to the control of essential molecular activities for stem cell, such as quiescence, activation, differentiation, and apoptosis [119,120]. Unfortunately, in geriatric satellite cells, autophagy is decreased resulting in the accumulation of damaged proteins and organelles, especially mitochondria, and increased levels of reactive oxygen species (ROS) causing DNA damage, senescence entry, and stem cell exhaustion [121], emphasizing the importance of proper autophagy process in preventing muscular atrophy [122].

Autophagy is aberrant in aged muscles. In addition, excessive lysosomal proteins, called lipofuscins, have been detected in rodents [123]. Defective autophagy has been reported in sarcopenia [124,125], in particular, Atg7 KO mice have been characterized as an animal model of sarcopenia [126]. In sarcopenic muscle, reduced autophagic flux promotes the transition to M1-proinflammatory macrophages, as well as the production of inflammatory cytokines and apoptosis [35]. A recent study indicates that the mTORC1 complex, containing the already-mentioned mTORC kinase, and mainly a regulatory-associated protein of mTOR called *raptor*, represents a critical therapeutic target in this disorder [127].

The selective autophagy called mitophagy, which is the autophagic process addressed to remove damaged mitochondria [128], is particularly interesting since it is increased in rodent models of sarcopenia [129]. The association between sarcoplasmic reticulum and mitochondria to provide an efficient calcium flux during contraction is dramatically affected in aged muscles and precedes sarcopenia [130]. Remarkably, in large proximal muscles, such as vastus lateralis, mitochondria alterations and accumulation of lysosomes not enveloping mitochondria in ragged red fibers have been described in patients [60].

The major molecular markers of mitophagy are Parkin and PTEN-induced putative kinase 1 (PINK1). This last kinase links to parkin and stimulates mitophagy to dismantle damaged mitochondria with altered mitochondrial transmembrane potential. Another Parkin-independent mechanism relies on other receptors, such as NIX or FUNDC1, which directly link the LC3 promoting mitophagy [61]. However, Parkin is required for exercise-induced mitophagy in muscle [131] and its overexpression protects aged muscle mass and strength [132].

Mitochondrial quality and activity are maintained in healthy active muscle by proper mitophagy [133]. Unfortunately, this process, defective in aging, generates accumulation [121] and dysfunction of mitochondria, predisposing to oxidative damage to mitochondrial DNA and ROS production [134]. Consequently, during aberrant mitophagy, apoptosis may occur leading to muscle atrophy and weakness [135]. Based on this evidence, analogously to what we have seen for autophagy, mitophagy manipulation might also be considered as a novel therapeutic opportunity in aging and sarcopenia [133].

Overall, autophagy seems to play a fundamental role in maintaining the mass and functionality of muscle by removing abnormal and dysfunctional organelles and misfolded proteins [136], whose accumulation in aged muscle is ascertained. This accumulation characterizing the aged muscle might be linked to many molecular and cellular mechanisms among which autophagy, autophagic changes, and defective signaling [137], for instance, the prolonged activation of mTORC1 [138], might be contributing to the progressive onset of sarcopenia, representing an interesting therapeutic target for consideration.

## 4. Polyamines: Sources, Synthesis, and Catabolism

Polyamines are natural caloric-restriction mimetic small cations, essential for cell growth, proliferation, differentiation, and tissue regeneration.

The sources of polyamine are derived from food (wheat germ, fermented soy, soybeans, aged cheese, mushrooms, peas, nuts, apples, pears, and broccoli) and from the production of intestinal microorganisms (microbiota, probiotics, and bacteria-derived), as well as cellular synthesis. Subsequently, polyamines can be resorbed by intestinal epithelial cells and distributed through systemic circulation [139].

Polyamines play a crucial role on DNA and RNA, act as antioxidants, modulate enzyme functions, as well as control apoptosis and the processes of learning and memory in the brain [140]. Remarkably, polyamine levels show circadian oscillations, with a frequency of approximately 24 h. Moreover, their cellular concentration leads to activation of a feedforward mechanism that affects the circadian rhythmicity, through the regulation of the interaction between the core clock gene repressors PER2 and CRY1 [141]. A central pacemaker in the suprachiasmatic nucleus (SCN) of the hypothalamus regulates the rhythmicity of the biological functions’ oscillation, both in animal and human. Circadian clock gene expression is significantly modified in the SCN and peripheral oscillators of aged animals [142], and this could be related to changes in spermidine levels during aging.

Indeed, polyamine levels are regulated by control mechanisms based on their synthesis, catabolism, and transport [143]. Specifically, ornithine decarboxylase (ODC) and S-adenosylmethionine decarboxylase (AdoMet-DC) regulate the polyamine biosynthesis. In contrast, spermidine/spermine-N^1^-acetyltransferase (SSAT), polyamine oxidase (PAO), and spermine oxidase (SMOX) regulate the polyamine catabolism [144].

Arginine, ornithine, and methionine are the polyamine forerunner.

Two synthetic polyamine pathways are identified: (I) The mitochondrial enzyme arginase converts the arginine in ornithine. Subsequently, the decarboxylation of ornithine allows for the production of putrescine. This process is mediated by the ODC; (II) L-methionine is converted to S-adenosyl-L-methionine (AdoMet), which is then decarboxylated by AdoMet-DC to produce decarboxylated AdoMet (DC-AdoMet). DC-AdoMet acts in the conversion process of putrescine to spermidine, and spermidine to spermine, which are both regulated by the spermidine synthase. The latter two mechanisms occur since decarboxylated AdoMet gives an aminopropyl group to putrescine or to spermidine [143].

In contrast, the polyamine catabolism reconverts spermine and spermidine.

Crucially, two different processes could mediate this conversion: (I) SSAT—PAO axis [143], and (II) spermine oxidase (SMOX) [139,144]. On the one hand, SSAT acetylates both spermine and spermidine. Subsequently, in the peroxisome, PAO reconverts the acetyl-spermine into spermidine, and the acetyl-spermidine into putrescine.

On the other hand, in the cytoplasm, SMOX reconverts spermine into spermidine. Byproducts of these oxidations include hydrogen peroxide (H_2_O_2_) and acetaminopropanal. As reported by Cervelli et al. (2018), H_2_O_2_ production inhibits myotube formation and markedly decreases myogenic expression [144]. Therefore, a decrease in SMOX activity would result in low H_2_O_2_ levels, which would enhance cell differentiation [144,145]. On this basis, impaired SMOX downregulation might result in defective myotube differentiation, leading to neoplastic transformation and promotion of several cancer cell types.

Polyamine transport is important for their regulation.

Minois et al. [143] demonstrated, in yeast, that phosphorylation and dephosphorylation processes control polyamine transport and that the latter are energy dependent. The DUR3, SAM3, GAP1, and AGP2 proteins are important for polyamine uptake across the plasma membrane. TPO1 to 4 excrete polyamines at acidic pH, but absorb them into the yeast cell at pH 8. In the Golgi complex and post-Golgi vesicles, TPO5 is a protein involved in polyamine excretion. Moreover, the authors showed that in Escherichia coli, a spermidine preferential system and a putrescine-specific system belong to the ATP-binding cassette (ABC) transporter family. In this context, four carriers play an important role. PotA to D for spermidine and PotF to I for putrescine. PotE and CadB are two other carriers, which absorb polyamines at neutral pH and excrete them at acidic pH. Finally, MdtII is identified as a spermidine excretion protein [143].

However, no studies have yet demonstrated the presence of polyamine transporter in mammals [143].

The most studied polyamines are the spermine and spermidine [146,147].

On the one hand, spermine contributes to maintaining the cellular physiological status, acting as a regulator of DNA synthesis, cellular proliferation, and second messenger in cellular signaling, and plays an important role in brain function [145].

On the other hand, spermidine is a natural polyamine involved in several important molecular and biological processes, such as DNA stability, transcription, translation, apoptosis, cell proliferation, differentiation, and survival [143,148].

## 5. Polyamines: A Potential Therapeutic Target in Aging and Related Pathologies

Dysregulation of polyamines and their metabolic enzymes have been associated with several pathologies [149].

Many studies documented a relationship between polyamine levels, drug response, apoptosis, and the etiology of adverse pathological conditions, including cancer [140]. Sanchez-Jimenez et al. (2019) showed that altered polyamine metabolism was related to neurodegenerative diseases, mental retardation, psychiatric disorders, and inflammation [150]. In detail, in neurological functions, polyamines should play a role as neurotransmitters or modulators of neurotransmission. The effects of polyamine on trained learning and memory, and the neuroprotective rules of spermidine supplementation have been recently reviewed [139,150,151]. Moreover, a loss of polyamine homeostasis seems to be associated with psychiatric disorders, such as depression [152], anxiety [153], schizophrenia, and epilepsy [154]. Regarding the immune responses, polyamine could act as immunostimulatory in immune cell differentiation, activation, recruitment, and as modulator of the inflammatory reaction [139,150,151,155]. Furthermore, polyamines are essential for the treatment of metabolic syndrome, obesity, and type 2 diabetes [139]. In addition, polyamine levels decrease with age in many organisms, as well as in humans [156,157,158]. In fact, the levels of the two main studied polyamines, spermine and spermidine, were significantly altered in skeletal muscles of aged mice [159].

Remarkably, spermine and spermidine are natural autophagy-inducers [156] and have anti-aging effects. A study showed that 2 weeks of spermidine administration in elderly mice reversed the age-associated defect in autophagy and mitophagy in muscle stem cell, and enhanced muscle regeneration [121,160]. The suppression of stem cell senescence by spermidine is dependent on autophagy restoration in satellite cells since spermidine promotes mitochondrial function. These stemness-enhancing effects ensure muscle regeneration and reduction in myopathy [139].

Due to their effect on skeletal muscle atrophy and hypertrophy, the polyamines’ role in skeletal muscle disease, such as sarcopenia, is evident as reported in a recent review [144].

Furthermore, as a geroprotector, spermidine decreases markers of age-related oxidative damage in mice [156], preserves mitochondrial function, holds anti-inflammatory properties, and prevents stem cell senescence [161]. In particular, the anti-inflammatory properties are exerted by several mechanisms that directly result on the reduction in the expression of pro-inflammatory cytokines, as recently well-reviewed by Ni et al. [162]: (I) Inhibition of accumulation of ROS, (II) decrease in the expression levels of tumor necrosis factor-α (TNF-α), (III) suppression of the translocation in the nucleus of nuclear factor-kB (NF-kB) p65 subunit [163], (IV) inhibition of the expression of IL-18 and IL-1β [162,164], and (V) attenuation of receptor interacting protein (RIP1) deubiquitination in macrophages, chondrocytes, and synovial tissue of osteoarthritic mice model [165,166].

Moreover, their supplementation contrasts the age-associated disruption of circadian rhythm in mice [167]. In addition, increasing polyamine levels through dietary supplementation may contribute to longevity [144,156]. It has been demonstrated that polyamines reduce age-related damage in short-lived mice [168] and counteract cardiovascular disease, neurodegeneration, and cancer [139,145,154]. Based on this evidence, polyamine (as supplementation or nutritional intake) could represent a potential preventive agent in the fight against age-derived muscle disability. Nonetheless, as reported in a recent review, the effects of polyamines in human and animal health can be dualistic depending on the cellular health state [151], which can be beneficial in healthy cells by boosting physiological processes to reduce aging- and stress-induced responses or detrimental in unhealthy cells under pathological conditions, such as cancer and neurological disorders [140,169], as above-mentioned. In fact, the functions exploited by polyamines in the complex lead toward the control of cellular death and life [143]. Cell death regulation, autophagy regulation, cell differentiation and reprogramming, and protein synthesis are all effects that might induce or modulate polyamines and trigger cellular changes for the promotion of both their growth and death via different mechanisms. Moreover, reversed reactions can be induced in cytoplasm and nucleus, making the understanding of the interaction of polyamines with aging, stress, and disorders even more complex to understand [169]. Although it seems that polyamines, in particular, spermidine have beneficial effects in promoting longevity in healthy organisms [156], we are still far away from comprehending their potential in age-related diseases, which remains putative in humans since this has only been investigated in non-human models to date.

Finally, spermidine received great attention for its potential to target some mechanisms behind autophagy induction, whose role might be a good target for increasing longevity and perhaps to prevent some diseases based on the exacerbation of the aging process, for example, sarcopenia in aged muscle.

## 6. Spermidine: An Autophagy Inducer

Numerous studies underline the relationship between aging, spermidine, and autophagy, indicating that spermidine is an important and specific inducer of autophagy [170]. The spermidine supplementation proved the effects of autophagy induction in some different models, such as yeast, flies, and mice [158,171]. Some proposed mechanisms of autophagy induction by spermidine in C2C12 mouse myoblast rely on the AMPK pathway, which has a double effect to upregulate the ULK complex (ULK1) as well as to inhibit mTORC1 activity; both events enhance the activation of the very first step (induction) [98,172]. In human cancer cell lines and non-transformed murine embryonic fibroblast, spermidine was found to stimulate the autophagy-associated lipidation of LC3, confirming the role of inducing autophagy [173]. In addition, Pietracola et al. (2015) proved the autophagic induction played by spermidine in cell-free systems. The authors concluded that spermidine inhibits acetyltransferases, in particular, E1A-binding protein p300 (EP300), the lysine acetyltransferase also known as NAT5, which irreversibly catalyzes the N-terminal acetylation of methionine residues (an irreversible post-translational modification) [174]. EP300 has been described as “an endogenous repressor of autophagy” [174] and its activity counteracts that of other autophagic inducers as the members of the AMPK family [175]. In fact, EP300 acetylates the core proteins of the autophagic machinery, for instance, ATG proteins [176], LC3 [177,178], and Beclin1 [179], thereby interfering with their pro-autophagic function. In addition to the aforementioned effects, the inhibition of EP300 strongly decreases p62/SQSMT1 and LC3-binding proteins important for leading the autophagic flux [178] and, although this still requires in-depth investigations, it has some molecular link with the inhibitory activity of mTORC1 on autophagy [180].

**Figure 2:**
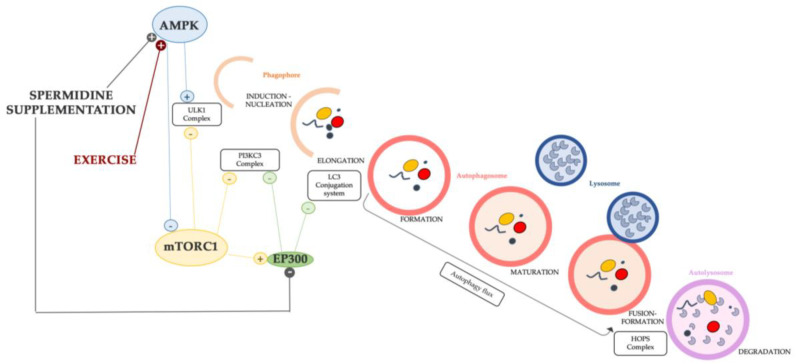
Schematic diagram of the mechanism of autophagy induction and inhibition implemented by spermidine and exercise. The link between the mTORC1 pathway and EP300 might be crucial for restoring a balanced autophagy in sarcopenic muscle.

As determined by spermidine, the effects of the above-mentioned mechanisms in regulating the autophagic process are synthetized in Figure 2. Some mechanisms involved in the induction of autophagy are also modulated by other compounds and/or factors, for example, physical exercise which is another inducer of autophagy in aged skeletal muscle (Figure 2), as it will be addressed in the next paragraphs.

## 7. Exercise-Induced Autophagy Activation

Muscle maintenance is based on protein synthesis and degradation, as well as the turnover of contractile proteins. Autophagy promotes the clearance of damaged proteins and other cellular components [69]. Moreover, the lysosomal autophagic pathway and the ubiquitin-proteasome pathway mediate the protein degradation in the skeletal muscle [122]. Recent studies based on animal models have demonstrated that physical exercise also induces autophagy and mitophagy processes [53,58], which can activate stem cell function, regulate skeletal muscle remodeling and homeostasis, and promote aged skeletal muscle repairing and hypertrophy. All these events decrease the regeneration of impaired muscle fibers related to aging [135]. During exercise, the catabolic pathways are accelerated to supply energy and is a substrate for muscle contraction. Since skeletal muscle is considered as a metabolic organ, autophagy could be induced by different forms of exercise training [181]. Exercise-induced autophagy activation leads to satellite cell activation, muscle mass maintenance, and muscle adaptation. Several studies suggest that exercise training, by activating the autophagy process, leads to an increase in muscle mass and function [182]. For instance, 8 weeks of treadmill training, for 40 min 5 days per week upregulated the age-related attenuation of autophagy processes in mice, as proven in the study by Kim et al. (2013) [122]. Expression of microtubule-associated protein light chain 3-II (LC3-II) is a marker of autophagy. Its lower levels indicate a reduced autophagic activity. Starting from lower levels of LC3-II protein expression, in older mice compared to young mice, the authors found increased levels of LC3-II protein after treadmill training in trained versus untrained older mice.

The exercise-induced autophagy is promoted and regulated by the balance of two mechanisms related to the AMP-activated protein kinase (AMPK) and protein kinase B (AKT) functions. In fact, AMPK and AKT are simultaneously stimulated by physical exercise and muscle contraction [183].

On the one hand, AMPK can induce the phosphorylation of FOXO, which as mentioned above, is an important transcription factor in different autophagic pathways that stimulates mitophagy and transcriptional activation of autophagy-related genes which oversee protein degradation [184]. FOXO family members (FOXO1, FOXO3, FOXO4, and FOXO6) are transcription factors that bind to the promoter regions and transactivate the expression of autophagy genes to induce autophagy [185]. They may also interact directly in the cytoplasm with autophagy proteins (Atg7) to regulate autophagy or employ epigenetic mechanisms (histone modifications and microRNAs) to control autophagy activity [185]. The activation of FOXO induced by AMPK through the post-translational modifications led to its translocation from the cytoplasm to the nucleus. Once activated, FOXO promotes the autophagy by transactivating the expression of the genes encoding autophagy proteins involved in the multiple stages of the autophagic process *induction*, acting on unc-51-like kinase (ULK)-1 and 2; *nucleation*, acting on Becn1 and Atg14; *elongation*, acting on Map1lc3b, Gabarapl, and Atg4; *autophagosome–lysosome fusion*, acting on Tfeb and Rab7 [185].

On the other hand, AKT inhibits FOXO through direct phosphorylation or indirectly activates the mammalian target of rapamycin (mTOR), which contrasts the FOXO migration into the nucleus, and thus inhibits the autophagy [186]. Moreover, mTOR stops the initiation of autophagosome formation acting on the ULK1 [182].

All these factors contribute to an adequate maintenance of metabolic processes and mitochondrial control in skeletal muscle. An appropriate regulation of exercise-induced AMPK and AKT can lead to the balance between these processes during aging, delaying, or alleviating sarcopenia [57,69].

The activation of exercise-induced autophagy in skeletal muscle depends on several factors, such as intensity and duration of physical activity and type of muscle fibers involved. For example, it seems that 15 months of resistance exercise enhance the autophagy pathway and suppress the sarcopenic phenotype in muscle of training mice compared to sedentary controls [187]. Moreover, 9 weeks of resistance exercise reveal decreased LC3-II/LC3-I ratio and reduced p62 protein levels, a classical receptor of autophagy, as well as increased autophagy regulatory proteins, including Beclin1, autophagy-related genes (ATG) 5/12/7, and lysosomes, thereby effectively preventing muscle loss and increasing muscle strength [188]. Therefore, regular exercise training can increase the level of autophagy through p62 protein and improve muscle function and strength. In addition, resistance exercise activates the phosphoinositide 3-kinase (PI3K)/AKT/mTOR, mTOR/ULK 1, and AKT/FOXO3 anabolic signal pathway, and the activation of AKT signaling by insulin or IGF-1 inactivates FOXO-dependent transcription. These signal pathways promote protein synthesis, reduce muscle protein breakdown, downregulate autophagy, inhibit protein degradation in skeletal muscle, and maintain muscular hypertrophy [189,190,191]. All of these factors can prevent age-related health problems, especially sarcopenia. Conversely, evidence suggested that endurance exercise promote AMPK and PGC-1α activity by improving mitochondrial function and skeletal muscle quality [56]. Moreover, endurance exercise has been shown to activate the AMPK/ULK1 signal pathway [54]. Endurance training activates AMPK and phosphorylates ULK1 in skeletal muscle, by inhibiting mTOR-mediated phosphorylation of ULK1.

Furthermore, Fan et al. (2017) demonstrated that endurance exercise can improve aerobic capacity by activating AMPK/FOXO3 signal pathway to delete damaged proteins and produce new energy substrates for skeletal muscle contraction and metabolism [183]. Based on these processes, it can be argued that endurance exercise can induce autophagy by activating AMPK/ULK1 and AMPK/FOXO3 signal pathways, promote the degradation of dysfunctional proteins in skeletal muscle, and generate energy substrates for skeletal muscle contraction. Both resistance and endurance exercises may prevent age-related muscle wasting and can be a treatment for sarcopenia through the improvement of autophagy [182]. Many studies have investigated the relationship between resistance or endurance exercise, autophagic processes, and longevity but there is limited evidence regarding the role of combined exercise training. However, combined exercise training can enhance protein synthesis in skeletal muscle through mTOR/p70S6K phosphorylation, but substantial improvements in skeletal muscle hypertrophy and strength have not been demonstrated [135]. The sustainability of autophagy activation is debatable in response to chronic exercise training [192,193,194]. It affects the protein turnover systems in skeletal muscles through two major signaling pathways. The first is the mechanistic target of the rapamycin complex 1 (mTORC1) pathway, associated with protein synthesis and hypertrophy. The second is the FOXO belonging to a family of transcription factors implicated in the regulation of protein breakdown and mitochondrial turnover [195]. Moreover, the FOXO transcription factors regulate the elimination of dysfunctional organelles, the ubiquitin-proteasome, and the autophagy-lysosome pathways. The latter two, as mentioned above, mediate protein degradation in skeletal muscle. Furthermore, acute exercise stimulates the autophagy process and increases insulin sensitivity in skeletal muscle of aged mice [55].

## 8. Effects of Spermidine Supplementation and Exercise in Skeletal Muscle

The findings discussed hereafter refer mainly, but are not limited, to those presented in the past 5 years by preclinical in vivo studies that focused on the role of the two treatments, spermidine supplementation and exercise, alone or combined, on autophagy in the skeletal muscle. In particular, only studies focusing on murine models (with muscle defects or aged) were considered in Table 1.

Concerning physical exercise, it is known that its regular administration generates a stimulus for muscle adaptation, decreasing the loss of skeletal muscle [195]. Additionally, in a sample of veteran soccer players (64–71 years) with at least 10 years of sporting activity, a higher polyamine level than age-matched untrained subjects was found [196]. Autophagy attenuation, a feature of the aging process, has been shown to be reduced by exercise. Regular exercise has been demonstrated to decrease DNA fragmentation and apoptosis in skeletal muscle [183]. Furthermore, exercise may decrease endoplasmic reticulum stress and apoptosis by activating the AMPK-dependent autophagy pathway [197].

Halling et al. [53] identified that mitochondrial fragmentation is a mechanism that contributes to aging-induced mitochondrial dysfunction in skeletal muscle. In fact, in a mitochondrial impairment murine model (PGC-1α knockout), representing an aged muscle model, it was evident that exercise training prevents mitochondrial fragmentation by suppressing mitochondrial fission protein expression via PGC-1α with concomitant modulation of autophagy. Later, these findings were also supported by Erlich et al. [56], who reported that TFEB along with PGC-1α positively regulate mitochondrial biogenesis in muscle as a result of exercise.

Moreover, direct evidence was provided for skeletal muscle by Laker et al. and Wang et al. [54,59], who proved that exercise respectively induced mitophagy in C57BL/6J mice and autophagy in ICR/CD-1 mice, both conventional murine models, through AMPK signaling. In addition, in the same aged murine model (C57BL/6J), not only ULK1 was found to increase, but also Atg5, Atg7, and LC3-II when exercise was administrated [55]. The latter LC3-II was found to increase when exercise was applied even to a sedentary murine model (Sprague–Dawley) [57].

Finally, Zeng et al. [58] elegantly demonstrated that exercise interventions modulate the AKT/mTOR, AKT/FOXO3a, and AMPK signaling pathways, and thus induce autophagy, maintain mitochondrial quality control, and suppress E3 ubiquitin ligase in skeletal muscle of aged rats (Wistar rats), implying that they all have an implication in mitigating sarcopenia upon exercise treatments.

Concerning spermidine, it is well-known that its administration could reactivate the autophagic flux in muscle [198] and its role on this system was elegantly presented by Chrisam et al., who demonstrated its effectiveness as an autophagic inducer in ameliorating myopathic muscle defects by acting on AKT [170]. In fact, on a genetically determined muscle disease mouse model (Col6a1^−/−^), spermidine proved the reduction in AKT phosphorylation, and thus the promotion of the translocation of FOXO transcriptional factors into the nucleus, with consequent increase in autophagic flux. This mechanism led to the attenuation of the skeletal muscle atrophic defects on animal models [175], placing the foundations for the possible use of polyamines for re-activating the autophagy in muscle and related pathologies. In a recent study, the administration of spermidine proved the enhancement of autophagy in satellite cells of skeletal muscle of adult pathological-free mice by inhibiting the protein expression of EP300, in addition to the inhibition of myostatin (MSTN) through repression of activin A receptor type 2B (ACVR2B) [160]. Although the authors proved that spermidine effects are exerted for both pathways and promote quiescent satellite cells activation; therefore, the number of myoblasts in adult skeletal muscle is increased. However, the authors have also stressed the importance of potentially considering the adverse side effects due to a long-term administration [160].

These results suggest that exercise and spermidine may share mediators acting on similar pathways in autophagy and related processes involved in muscle maintenance.

Alterations in these processes are displayed in physiological condition, such as aging, and in diseases, such as sarcopenia and other pathologies related to muscle atrophy. Aging is characterized by a gradual deactivation of the autophagic processes during age, decreasing the efficiency of protein degradation and the clearance of damaged cellular organelles [144]. Autophagy attenuation that occurs with aging could be reduced by exercise training and spermidine supplementation, and more importantly, as a combined treatment. In fact, the combination of the two treatments was investigated by Fan et al. [183] on a normal murine model aged by the D-gal treatment. D-gal-treated rats displayed a greater reduction in the ratio of gastrocnemius muscle weight to body weight compared to the control group. On this deficient autophagic signal model, spermidine and exercise increased Beclin1, LC3-II, and p62 via AMPK signaling pathway [183]. In addition, the lower level of the ratio of gastrocnemius muscle weight to body weight inducted by D-gal was contrasted by spermidine coupled with exercise-reaching values comparable to the control group [183]. Table 1 displays the studies that investigated the role of spermidine supplementation, exercise effects, and their combined action on autophagy and mitophagy in mice muscle.

In support of the hypothesis that the combination of physical activity and spermidine supplementation could have positive effects on health, Schipke et al. (2019) showed that the two treatments combined lead to a lower body weight, and significantly affect the taxonomic composition of the gut microbiota, compared to activity or spermidine alone, highlighting further beneficial effects exerted by the two treatments combined [199].

**Figure 3:**
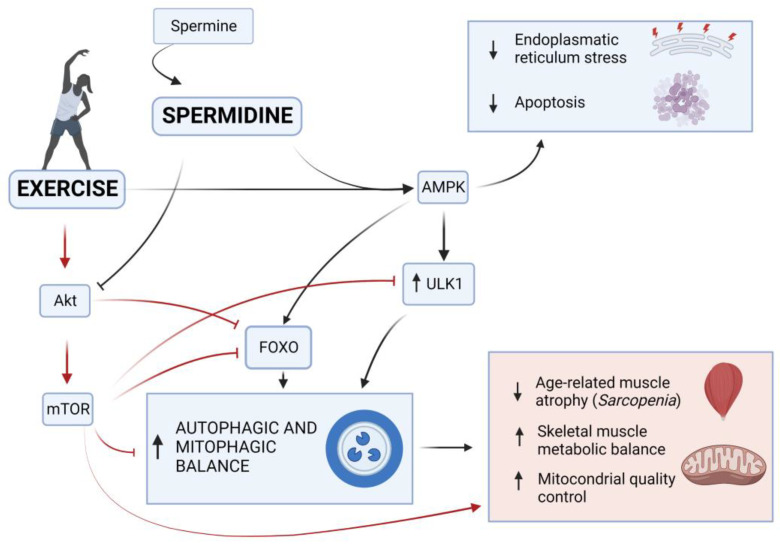
Role of exercise and spermidine in healthy aging. Exercise and spermidine play an important role in healthy aging through the regulation of muscle maintenance by acting on the autophagic AMPK and AKT pathways. Exercise-activated AMPK can induce the phosphorylation of FOXO, stimulating mitophagy and transcriptional activation of autophagy-related genes, which oversee protein degradation. AMPK can also upregulate ULK1, promoting the induction phase of the autophagic process. In contrast, exercise-activated AKT inhibits FOXO through direct phosphorylation or indirectly activates the mTOR, which contrasts the FOXO migration into the nucleus inhibiting the autophagy. The mTOR also stops the initiation of autophagosome formation acting on the ULK1. On the other hand, spermidine supplementation promotes the AMPK-induced autophagy and blocks AKT-inhibiting activity on autophagy. All these factors contribute to an adequate maintenance of metabolic processes and mitochondrial control in skeletal muscle during aging, delaying, or alleviating pathological conditions, such as sarcopenia (created with BioRender.com).

The effects that spermidine and exercise share on the regulation of the autophagic process in muscle that we have seen until now push us to consider both as promising interventions for slowing aging processes in age-related muscular skeletal disorders as sarcopenia. In addition, as both represent ideal candidates for future clinical evaluations, their combination could properly activate the autophagic process, maintaining the skeletal muscle and delaying its senescence [191] in a synergetic way, as illustrated in Figure 3. In addition, their synergic effect might allow for the optimization of a safe dose of spermidine, and thus the avoidance of the toxicity and negative side effects that have been linked and assumed for high doses and long-term uses, as controversially debated in literature [200].

## 9. Conclusions

Tuning autophagy as a potential treatment for age-related musculoskeletal diseases is of utmost importance, but raises many critical considerations.

First, in our opinion, the presence of comorbidity in the elderly, such as atherosclerosis, neurodegeneration, and diabetes, makes it very difficult to extrapolate data obtained in rodents. Fritzen et al. (2016) reported differences in autophagy regulation and estimation of the autophagic flux in humans vs. rodents [201]. Indeed, exercise-induced autophagosome production is different in humans and rodents and is strictly dependent on the type of skeletal muscle and intensity or duration of the exercise. Moreover, body metabolism and circadian rhythmicity are completely different since rodents are mainly active at night as opposed to humans [202]. Nevertheless, it is important to remark that exercise-induced autophagy and mitophagy processes are able to promote the repair and hypertrophy of aged skeletal muscle [135,182]. Therefore, researchers need to consider differently humans and animals in future studies.

Second, in this study, we did not examine gut-muscle axis influence on sarcopenia and autophagy [203]. Intestinal microbiome can synthesize polyamines, reinforcing their presence in the body, but dysbiosis and low inflammation, common in the elderly, may affect the efficacy of polyamine supplementation and transport.

This important point is still relatively unexplored, even if recent evidence indicates that dystrophic mdx/mdx mice supplemented with short-chain fatty acids by gut metabolites rescued muscle strength and autophagy [204]. Moreover, reciprocal influence between gut microbiota products and autophagy and mitophagy in rodent muscles has been reported by Ref. [205].

Third, increased polyamine catabolism provides some products and enzymes with critical side effects, implicated in the development of cerebral ischemia, traumatic diseases, and cancer [206]. Indeed, agents able to block the polyamine synthetic pathway by the inhibition of ODC enzyme, have been used to avoid recurrence in colorectal cancer [207]. However, if polyamines could act as oncometabolites, their effect is dose-dependent and must be strictly considered in long-term daily treatment.

Daily doses of 1.2 mg spermidine, 0.6 mg spermine, and 0.2 mg putrescine, assumed for 3 months in wheat-germ extracts, are safe and well-tolerated in old patients with subjective cognitive decline at risk of developing dementia, even if muscle atrophy has not been evaluated [208]. However, 0.9 mg of spermidine per day for 12 months in similar patients ameliorated verbal memory deficits [209].

The main aim of the current review was to discuss the effects of polyamines and physical activity on musculoskeletal diseases. To the best of our knowledge, while few studies [160,170] reported that spermidine supplementation in mice models improves autophagy in aged or defected muscle, more numerous studies revealed that exercise intervention promotes the autophagy and mitophagy processes in the skeletal muscle through the regulation of muscle remodeling and homeostasis, and by promoting aged skeletal muscle repairing and hypertrophy [53,54,55,56,57,58,59].

Finally, the combination of spermidine supplementation and regular physical exercise is still little investigated as only one study reported data to date [183]. Their combined effect could have positive effects on reactivating the autophagic process flux, maintaining the skeletal muscle mass, and delaying its senescence [183]. These results suggest that exercise and spermidine may share mediators acting on similar pathways in autophagy and related processes involved in muscle maintenance. Therefore, the established geroprotective effect of spermidine supplementation and regular practice of exercise might also be promising to prevent or improve age-related musculoskeletal diseases. Based on these findings, future research developments, in terms of preclinical and clinical evaluations, may stress the empirical role that polyamine and physical activity play synergically in alleviating sarcopenia or age-related muscular skeletal diseases. However, we wish to note that an optimization of both treatments in terms of dose and duration is essential in order to achieve successful and beneficial autophagic effects that avoid the advent of paradoxical contrary effects, an aspect that should be well-considered in future researches.

## Figures and Tables

**Table 1:** Recent studies targeting autophagy and mitophagy with different interventions.

	Intervention	Model	Results	Reference
Autophagy and Mitophagy	Spermidine	8-week-old specific pathogen-free male C57/BL mice.	Induced muscle atrophy and improved autophagy in muscle stem cells.	Zhang et al., 2018 [160]
	Spermidine and Exercise	4-month-old Sprague–Dawley male rats. D-galactose-induced aging rats with skeletal muscle atrophy.	Increased autophagy.	Fan et al., 2017 [183]
	Exercise	3-month-old until 15-month-old female whole-body PCG-1α knockout and wild type littermate mice.	Maintained mitochondrial function and counteracted the aging-induced increase in autophagy proteins.	Halling et al., 2017 [53]
	Exercise	10/12-week-old male C57BL/6J mice.	Induced mitophagy in skeletal muscle regulated through the AMPK-ULK1 signaling axis.	Laker et al., 2017 [54]
	Exercise	3-month-old and 24-month-old male C57BL/6J mice.	Induced autophagy.	Lenhare et al., 2017 [55]
	Exercise	4/5-month-old male and female PCG-1α knockout and wild type mice.	Induced transcription factor EB (TFEB) and activation in a PCG-1α-dependent-manner in skeletal muscle. Induced mitophagy and expression of genes involved in autophagy coordinated by TFEB.	Erlich et al., 2018 [56]
	Exercise	6-week-old male Sprague–Dawley.	Increased autophagy and mitochondrial function in skeletal muscle.	Li et al., 2018 [57]
	Exercise	21-month-old Wistar rats.	Increased autophagy via mTOR, FOXO3a, and AMPK pathways.	Zeng et al., 2020 [58]
	Exercise	8-week-old male ICR/CD-1 mice.	Increased AMPK-related autophagy activation in skeletal muscle.	Wang et al., 2022 [59]

## Data Availability

Not applicable.

## Abbreviations

ABC transporters	ATP-binding cassette transporters
ACVR2B	Activin A Receptor Type 2B
AdoMet-DC	S-adenosylmethionine decarboxylase
AKT	protein kinase B
AMPK	adenosine monophosphate-activated protein kinase
ATG	autophagy-related genes
BCL2	Beclin2
Cad	cadaverine
Dc-AdoMet	decarboxylated AdoMet
D-gal	D-galactose
EWGSOP2	European Working Group on Sarcopenia in Older People 2
FOXOs	Forkhead box O
HOPS	homotypic fusion and protein sorting
IGF-1	insulin growth factor 1
IGF-1	insulin growth factor 1 receptor
IL-6	interleukin 6
IL-18	interleukin 18
IL-1β	interleukin 1β
LC3-I	microtubule-associated protein light chain 3-I
LC3-II	microtubule-associated protein light chain 3-II
MSTN	myostatin
mTOR	mammalian target of rapamycin
mTORC1	mTOR Complex 1
MYH	myosin heavy chain
NF-kB	Nuclear Factor-kB
ODC	ornithine decarboxylase
p62/SQSTM	sequestosome protein
PAO	polyamine oxidase
PGC-1α	peroxisome proliferator-activated receptor-gamma coactivator
PI3K	phosphoinositide 3-kinase
PINK1	PTEN-induced putative kinase 1
POT	Proton-dependent Oligopeptide Transporter
PTEN	phosphatase and tensin homolog enzima
RANKL	nuclear factor kappa B receptor activator ligand
RIP1	receptor interacting protein
ROS	reactive oxygen species
SCN	suprachiasmatic nucleus
SMOX	spermine oxidase
SSAT	spermidine/spermine-N^1^-acetyltransferase
TFEB	transcription factor EB
TNF-α	Tumor Necrosis Factor-α
ULK1	UNC51-like kinase
